# Genetic basis for probiotic yeast phenotypes revealed by nanopore sequencing

**DOI:** 10.1093/g3journal/jkad093

**Published:** 2023-04-27

**Authors:** Joseph H Collins, Lohith Kunyeit, Sarah Weintraub, Nilesh Sharma, Charlotte White, Nabeeha Haq, K A Anu-Appaiah, Reeta P Rao, Eric M Young

**Affiliations:** Department of Chemical Engineering, Worcester Polytechnic Institute, Worcester, MA 01609, USA; Department of Biology and Biotechnology, Worcester Polytechnic Institute, Worcester, MA 01609, USA; Department of Microbiology and Fermentation Technology, CSIR—Central Food Technological Research Institute (CFTRI), Mysore, Karnataka 570020, India; Bioinformatics and Computational Biology, Worcester Polytechnic Institute, Worcester, MA 01609, USA; Department of Chemical Engineering, Worcester Polytechnic Institute, Worcester, MA 01609, USA; Department of Chemical Engineering, Worcester Polytechnic Institute, Worcester, MA 01609, USA; Department of Biology, Brandeis University, Waltham, MA 02453, USA; Department of Microbiology and Fermentation Technology, CSIR—Central Food Technological Research Institute (CFTRI), Mysore, Karnataka 570020, India; Department of Biology and Biotechnology, Worcester Polytechnic Institute, Worcester, MA 01609, USA; Department of Chemical Engineering, Worcester Polytechnic Institute, Worcester, MA 01609, USA

**Keywords:** *Saccharomyces cerevisiae* strain KTP, *Issatchenkia occidentalis* strain ApC, probiotic traits associated genes, *FLO* genes

## Abstract

Probiotic yeasts are emerging as preventative and therapeutic solutions for disease. Often ingested via cultured foods and beverages, they can survive the harsh conditions of the gastrointestinal tract and adhere to it, where they provide nutrients and inhibit pathogens like *Candida albicans*. Yet, little is known of the genomic determinants of these beneficial traits. To this end, we have sequenced 2 food-derived probiotic yeast isolates that mitigate fungal infections. We find that the first strain, KTP, is a strain of *Saccharomyces cerevisiae* within a small clade that lacks any apparent ancestry from common European/wine *S. cerevisiae* strains. Significantly, we show that *S. cerevisiae* KTP genes involved in general stress, pH tolerance, and adherence are markedly different from *S. cerevisiae* S288C but are similar to the commercial probiotic yeast species *S. boulardii*. This suggests that even though *S. cerevisiae* KTP and *S. boulardii* are from different clades, they may achieve probiotic effect through similar genetic mechanisms. We find that the second strain, ApC, is a strain of *Issatchenkia occidentalis*, one of the few of this family of yeasts to be sequenced. Because of the dissimilarity of its genome structure and gene organization, we infer that *I. occidentalis* ApC likely achieves a probiotic effect through a different mechanism than the *Saccharomyces* strains. Therefore, this work establishes a strong genetic link among probiotic *Saccharomycetes*, advances the genomics of *Issatchenkia* yeasts, and indicates that probiotic activity is not monophyletic and complimentary mixtures of probiotics could enhance health benefits beyond a single species.

## Introduction

Probiotic microbes are present in many fermented and cultured products across diverse cultures. Several have been isolated and marketed as probiotic supplements. To be classified as a probiotic, a microbe must exhibit beneficial effects and be properly identified by phenotypic and genomic methods (Reuter *et al*. 2002). According to Qualified Presumption of Safety (QPS) developed by European Food Safety Authority (EFSA), definition of the taxonomy of a microorganism in feed and food application is a major safety parameter in the selection process (Sanders *et al*. 2010). In other words, both the phenotype and genotype of a microbe must be defined before it can be called a probiotic. We have recently established a whole genome sequencing (WGS) pipeline called Prymetime (Collins *et al*. 2021). This tool can achieve higher genome contiguity and accuracy than previous approaches. Therefore, applying Prymetime to probiotic yeast, WGS could improve taxonomic classification and provide insight into the genomic underpinnings of probiotic microbes. This could then be leveraged to genetically engineer targeted probiotic solutions for human health. Here, we apply Prymetime to 2 recently isolated yeast strains with beneficial properties.

Probiotic yeasts uniquely combine human health benefits and tolerance phenotypes that enable survival in the gastrointestinal tract (Kunyeit *et al*. 2023). They can produce beneficial metabolites and inhibit bacterial and fungal pathogens (Foligne *et al*. 2010; Kunyeit *et al*. 2021). They also can survive at human body temperature, withstand acidic and alkaline pHs similar to the digestive tract, and tolerate constituents of the digestive system like bile, gastric enzymes, and pancreatic enzymes (Tiago *et al*. 2009; Chen *et al*. 2010; Sornplang and Piyadeatsoontorn 2016). They also can adhere to gut epithelial cells (O'Mahony *et al*. 2005; Lohith and Anu-Appaiah 2018). Perhaps unsurprisingly, probiotic yeasts are frequently found in fermented foods and beverages. Although several yeasts are known to have probiotic properties, only *Saccharomyces boulardii* has been commercialized and is prescribed to control and prevent gastrointestinal complications (McFarland 2010; Offei *et al*. 2019).

WGS is the key to enabling yeast classification and domestication. For example, WGS of *S. boulardii* revealed that it was in fact a strain of *S. cerevisiae* with a few notable differences in galactose metabolism and flocculation genes (Khatri *et al*. 2013, 2017). This led to the expansion of genetic tools for *S. boulardii*, opening exciting possibilities for interrogating genotype-phenotype connections, and creating designer probiotics by CRISPR-mediated genome editing (Ansari *et al*. 2019; Durmusoglu *et al*. 2021).

Recently, 2 yeasts isolated from fermented beverages were shown to have probiotic effects against *Candida albicans* and non-albicans *Candida* strains (Lohith and Anu-Appaiah 2018; Kunyeit *et al*. 2019; Kunyeit *et al.* 2021). The first strain, KTP, was isolated from a fermented beverage made from coconut sap (coconut toddy). The second strain, ApC, was isolated from fermented apple juice. Both were shown to control *C. albicans* filamentation and adhesion properties (Lohith and Anu-Appaiah 2018; Kunyeit *et al*. 2021). These strains were also shown to limit adhesion, filamentation, and biofilm formation of several non-albicans *Candida* species, including *C. tropicalis*, *C. krusei*, *C. glabrata*, *C. parapsilosis*, and *C. auris* (Kunyeit *et al*. 2019). Yeasts isolated from fermented foods have been shown to be resistant to several stressors such as temperature, osmotic stress, oxidative stress, pHs and antimicrobials making them suitable for probiotic applications (Kunyeit *et al*. 2023). As evidence, 2 yeasts reported here also survived exposure to the harsh conditions of the gastrointestinal tract tested in simulated gastric and bile juices as well as attachment to Caco-2 epithelial cells, in *ex vivo* conditions (Lohith and Anu-Appaiah 2018). This current report explores the genomic underpinning of the beneficial traits for these two strains. Together these findings support the use of these yeasts as probiotics.

The genomic sequence of ApC reveals that it is *Issatchenkia occidentalis*, a non-*Saccharomyces* yeast with very few genomes reported. *I. occidentalis* strain ApC belongs to the *Pichiaceae* family and it exhibits phenotypic traits such as the ability to attach to the intestinal epithelia, tolerance to digestive juices, and production of extracellular enzymes. These traits make it a novel and attractive probiotic strain. A clear connection between the probiotic phenotypes of *I. occidentalis* ApC and its genotype is limited until more there is a more robust collection of non-*Saccharomyces* genomes. The novel genome presented here expands that collection and provides a benchmark dataset for future studies. In contrast, the genome sequence of KTP reveals that it is a strain of *S. cerevisiae* with a robust collection of reference genomes allowing whole genome analysis as well as investigation of individual genes previously implicated in probiotic phenotypes. Thus, this study expands the genomic information available for probiotic yeasts and sets a strong foundation for future comparative genomic and genetic studies enabled by WGS.

## Materials and methods

### Strain isolation and growth

The *S. cerevisiae* strain KTP and *I. occidentalis*, ApC, were originally isolated from coconut toddy and fermented apple juice, respectively. Both strains were grown in yeast extract peptone dextrose (YPD) media at 30°C overnight.

### Scanning electron microscopy

Twenty-four-hour-old yeast cells were harvested by centrifugation and washed 3 times with phosphate buffer saline (pH 7.4). Washed cells were then fixed in 2.5% glutaraldehyde and set aside overnight at 4°C. The fixed cells were washed with phosphate buffer saline and dehydrated by sequential exposure to 10, 30, 50, 80, 90, and 100% ethanol. The samples were coated with conductive layer of sputtered gold and examined under scanning electron microscope (LEO 435 VP LEO Electron microscopy, Cambridge, UK), and images were captured (Ruthu *et al*. 2014).

### Quantification of alcohol content

Strains KTP and ApC were inoculated in YPD media containing 10 and 20% glucose and incubated for 30 h at 28°C. Two milliliters supernatant was diluted with miliQ water (q.s. 50 mL) and distilled at 55°C. Fifteen milliliters of the distillate was collected in a graduated tube, and 2.5 mL of distillate was used to estimate alcohol content. Absorbance was read at 600 nm. For standard graph, range of 0–15% of absolute ethanol was used (Caputi *et al*. 1968; Vijayalaxmi *et al*. 2013).

### Genomic DNA isolation and sequencing

High-molecular weight genomic DNA was isolated based on a modified version of Promega's Genomic DNA Isolation Kit (Promega, A1120) 21. Nanopore reads were prepared for sequencing using the Rapid Barcoding Kit (ONT, SQK-RBK004). Illumina reads were prepared for sequencing using the Nextera DNA Flex Library Prep Kit (Illumina, 20018704) along with the Nextera DNA CD Indexes (Illumina, 20018707). Nanopore sequencing was started using the MinKNOW software from ONT with the default settings. The resulting fastq files were demultiplexed using EPI2ME (Metrichor, Oxford, UK). Illumina sequencing was started by using the native Local Run Manager on the iSeq 100 machine. A GENERATEFASTQ run was initiated and run with the parameters Read Type: Paired End, Read Lengths: 151, and Index Reads: 2. Reads were demultiplexed using the native software on the iSeq machine.

### De novo genome assembly and annotation

The *S. cerevisiae*, KTP, and *I. occidentalis*, ApC, genomes were assembled using the Prymetime (v0.2) pipeline (Collins *et al*. 2021), which uses both Nanopore and Illumina reads. The resulting assemblies were annotated using Augustus v3.2.3 (Stanke and Morgenstern 2005). Augustus requires a probable organism to be designated when processing a genome. Therefore, the probable organism for *S. cerevisiae* KTP was *S. cerevisiae*, while the probable organism for *I. occidentalis* ApC was *Pichia stipitis*.

### Genome assessment

The assembly statistics number of contigs, N50, assembly length, and GC percentage were generated using QUAST v5.0.0 (Gurevich *et al*. 2013). Completeness of the genome assemblies was calculated using BUSCO v4.0.6 with the *Saccharomycetes* database (Simao *et al*. 2015).

### Phylogenomics

The *S. cerevisiae* KTP strain was placed in the global *S. cerevisiae* phylogenetic tree using data from the *S. cerevisiae* 100 genomes project (Strope *et al*. 2015). The project used a set of 16 conserved regions to make a phylogenetic tree. KTP's corresponding 16 conserved sequences were extracted from the assembly using BLASTN (Johnson *et al*. 2008) and then concatenated into one fasta file. MAFFT v7.464 was used to create a multiple sequence alignment, with the phylogenetic tree constructed using the UPGMA setting (Katoh *et al*. 2019). The ApC strain was placed in the *Pichiaceae* family phylogenetic tree using BUSCO and the BUSCO-phylogenomics utility script (https://github.com/jamiemcg/BUSCO_phylogenomics). BUSCO v4.0.6 with the *Saccharomycetes* database was run on 53 publicly available *Pichiaceae* family genome assemblies, the ApC assembly, and the *S. cerevisiae* S288C assembly (outgroup). The BUSCO-phylogenomics script was used to construct a supermatrix alignment of highly conserved BUSCO families, followed by a Maximum Likelihood and Bayesian phylogenetic reconstruction on the supermatrix (McGowan *et al*. 2020). FigTree v1.4.2 was used to visualize the phylogenetic trees (http://tree.bio.ed.ac.uk/software/figtree/).

### Comparative genomics

The KTP assembly was compared to two other assemblies: *S. cerevisiae* S288C (Goffeau *et al*. 1996) and *S. boulardii* unique28 (Khatri *et al*. 2017). A list of probiotic-related proteins from *S. cerevisiae* S288C was obtained from the Yeast Genome Database (Cherry *et al*. 2012). BLASTP v2.6.0 was used to search for hits to these proteins in predicted proteomes of *S. cerevisiae* KTP and *S. boulardii* unique28. BLASTP was run with the 2/9 parameters “-task blastp -outfmt ‘6 qseqid sseqid pident qcovs’ -evalue 10e-5”. Protein alignments were run with Clustal Omega v1.2.4 with the default parameters (Sievers *et al*. 2011). ProgressiveMauve was used to align the S288C, KTP, and unique28 assemblies with the default parameters (Darling *et al*. 2010). Shared and unique orthologous groups among the S288C, KTP, and unique28 proteomes were identified using OrthoVenn2 (Xu *et al*. 2019). Proteins from the unique KTP orthologous group were first extracted and then BLASTP was used to identify the closest strain hit.

## Results and discussion

### Morphology

**Fig. 1.**
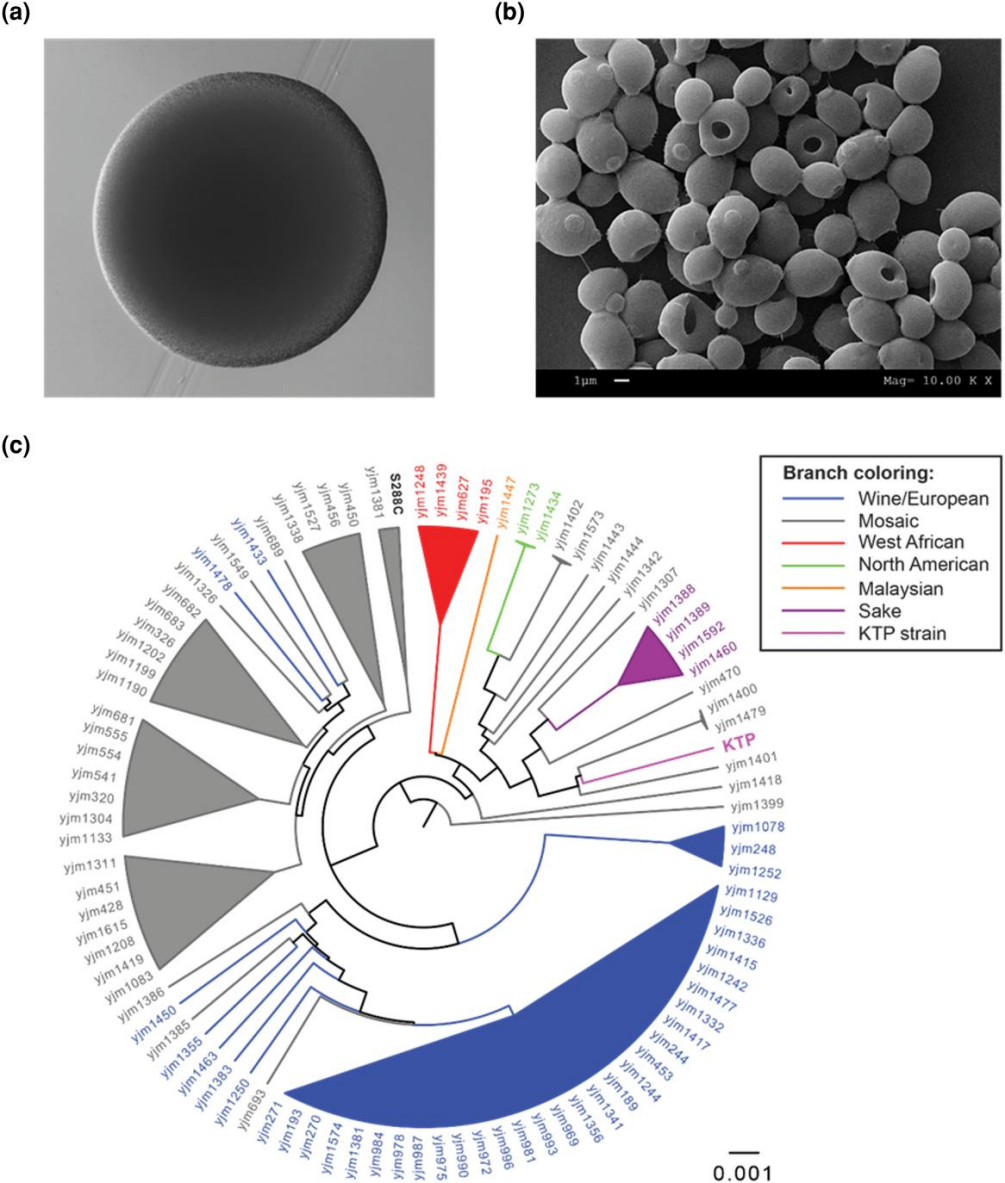
Analysis and placement of *S. cerevisiae* KTP. a) A colony of *S. cerevisiae* KTP imaged under a microscope after 24 h incubation on a YPD agar plate. b) Scanning electron micrograph of *S. cerevisiae* KTP. c) Global phylogenetic position of the KTP strain in comparison to other *S. cerevisiae* strains.

**Fig. 2.**
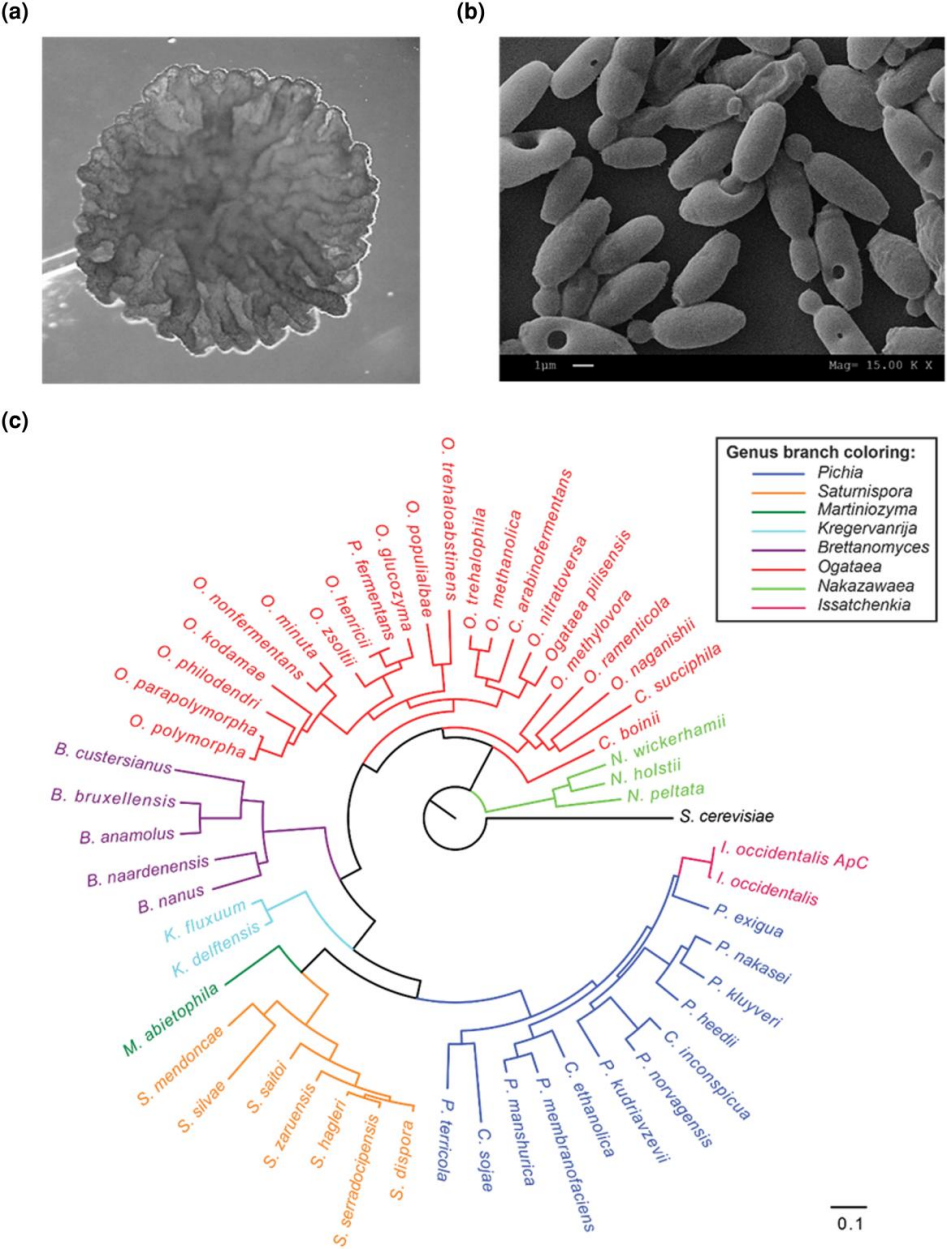
Analysis and placement of *I. occidentalis ApC.* a) A colony of *I. occidentalis* ApC imaged under a microscope after 24 h incubation on a YPD agar plate. b) Scanning electron micrograph of *I. occidentalis* ApC. c) Phylogenetic position of *I. occidentalis* ApC in the *Pichiaceae* family.

Before sequencing, we characterized colony and cell morphology of the 2 strains. Colonies of KTP are white and smooth while ApC forms rough colonies (Figs. 1a and 2a, respectively). Scanning electron microscopy shows that KTP cells are ovoid with evident buds, very similar to *S. cerevisiae* (Fig. 1b). ApC cells are more rod-like with buds occurring primarily at the end of the rods (Fig. 2b). These results corroborated the initial classification of KTP in *Saccharomyces* and ApC in *Issatchenkia*.

### Sequencing and genotyping with ITS sequences

Genomic DNA from KTP and ApC was isolated and sequenced using both short read (Illumina iSeq) and long read (Oxford Nanopore MinION) sequencing. The reads were assembled using Prymetime v0.2 (Collins *et al*. 2021) and annotated with Augustus v3.2.3 (Stanke and Morgenstern 2005). Genome assembly and annotation statistics are reported in Supplementary Table 1, and the raw data, assemblies, and annotation are published under BioProject PRJNA869102 for KTP PRJNA869107 for ApC (see Data Availability).

To initially classify the strains, we genotyped the strains using internal transcribed spacer (ITS) sequences. The method described by Kurtzman uses PCR of the ITS region followed by Sanger sequencing and BLAST to find related organisms. Here, we extracted the ITS regions of KTP and ApC from each genome assembly and input them into BLAST. The results are reported in Supplementary Table 1. The strain KTP is convincingly *S. cerevisiae*—the top 10 BLAST hits were all *S. cerevisiae* strains with percent identity above 99.5%. The species of the ApC strain was less clear—the top 10 BLAST hits were from several *Pichia* species. ApC was previously identified as *P. occidentalis* (now *I. occidentalis*) (GeneBank no.: KF551991.1) (Lohith and Anu-Appaiah 2018), but this initial genotyping observed that other *Pichia* species returned higher BLAST scores than *P. occidentalis.* These results provided enough information to further refine the phylogenetic position of each strain using the whole genome.

### Genotyping KTP and ApC using the whole genome

Since KTP was a candidate *Saccharomyces* strain, its phylogenetic position could be established using the *S. cerevisiae* 100 genomes project data as a reference (Strope *et al*. 2015). The *S. cerevisiae* 100 genomes project used a set of 16 conserved regions—one from each chromosome—to construct a global phylogenetic tree. To position KTP, these conserved regions were extracted from the KTP genome assembly and added to the 100 genomes project data (Fig. 1c). KTP is related to the Mosaic group, which has ancestry from two or more populations. KTP was closest to the yjm1400, yjm1479, and yjm1401 strains, which had significant ancestry from Sake, North American, and Malaysian strains. Unlike many of the strains tested in the 100 yeast genomes project, these three strains do not have any Wine/European ancestry.

ITS genotyping at least showed that ApC belonged in the *Pichiaceae* family. The *Pichiaceae* family yeasts are commonly found in spontaneous fermentation, as a result it commonly exists in several traditional fermented foods (Kunyeit *et al*. 2023). *Pichia* strains are used extensively in biotechnological applications, yet only a few such as *P. pastoris* and *P.* kudriavzevii are studied at the genomic level (Kuberl *et al*. 2011; Douglass *et al*. 2018). Furthermore, some *Piciaceae* family shows a significant genome similarity with other yeast species. For example, a comparative genome analysis of *P. kudriavzevii* revealed 99.6% genome identity with the pathogenic yeast, *C. krusei* (Douglass *et al*. 2018). These initial findings underscore the need for a larger pool of genomic information to better classify the *Pichiaceae* family. Yet, with the current available data, we were able to conduct a phylogenetic analysis with 53 publicly available *Pichiaceae* family genome assemblies (Piskur *et al*. 2012; Ravin *et al*. 2013; Strope *et al*. 2015; Riley *et al*. 2016; Roach and Borneman 2020). Without predefined regins for classification, the analysis on all strains was carried out with essential genes determined by BUSCO (Simao *et al*. 2015). To do this, a utility script was used to create a supermatrix alignment of highly conserved BUSCO families and produce a phylogenetic reconstruction (https://github.com/jamiemcg/BUSCO_phylogenomics). The reconstruction and visualization of the phylogenetic tree (Fig. 2c) indicates that ApC is in fact closest to *I. occidentalis*. This highlights the usefulness of accurate tools like Prymetime for analysis and annotation of whole genome sequences to corroborate and resolve the taxonomic classification of yeasts. While probiotic attributes of non-*Saccharomyces* yeasts such as *I. occidentalis* have been studied extensively using *in-vitro*, *ex-vivo*, and preclinical models (Kunyeit *et al*. 2023), there is not a suitable non-*Saccharomyces* reference strain to aid in the analysis of the ApC genome. However, we were able to further analyze *S. cerevisiae* KTP in comparison to the well-known commercialized *Saccharomyces* probiotic yeast, *S. boulardii,* as several genes and pathways have been implicated in its probiotic traits.

### Comparative genome analysis reveals conserved and unique KTP genome features

**Fig. 3.**
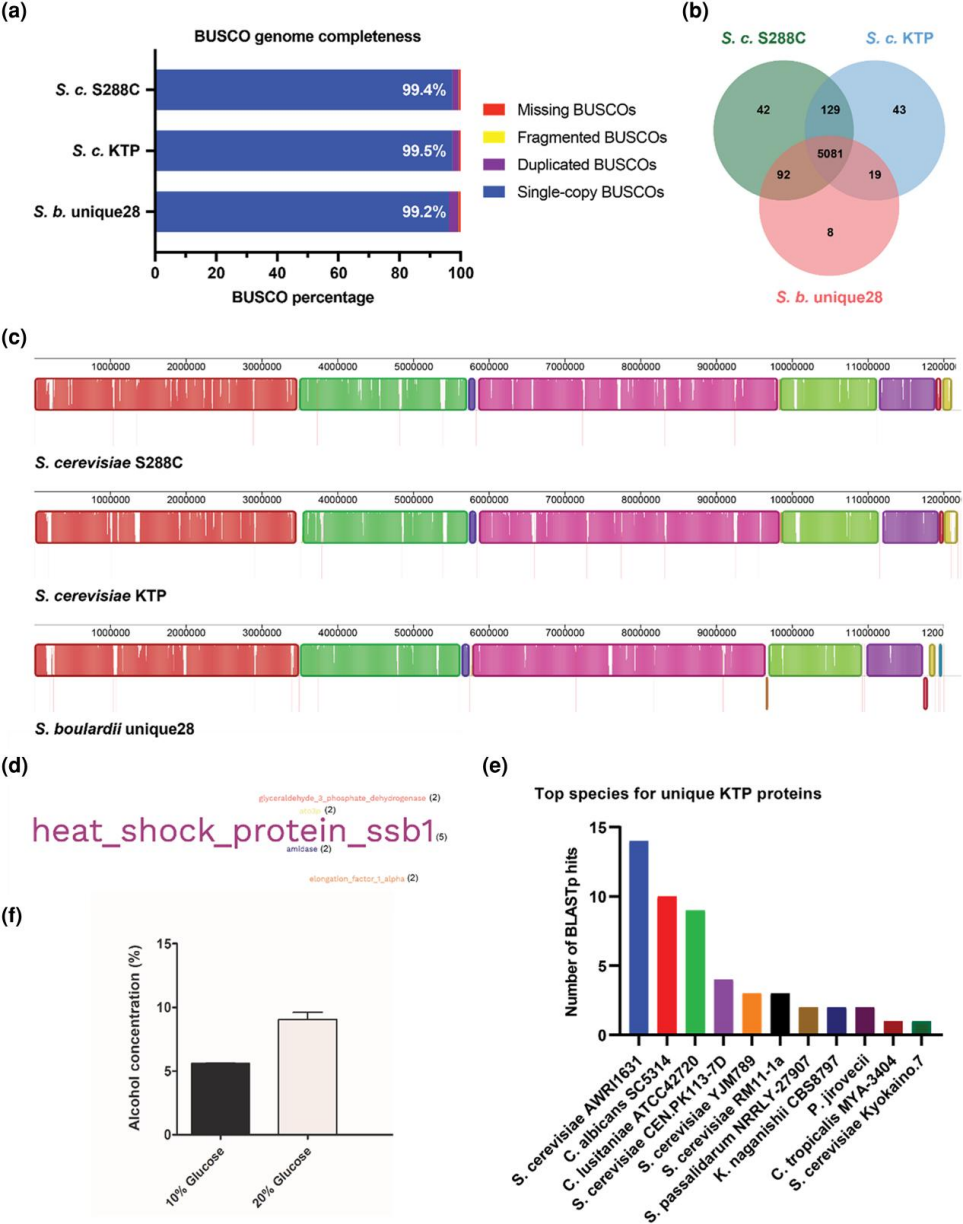
Comparing the genomes of *S. cerevisiae* S288c, *S. boulardii* unique28, and *S. cerevisiae* KTP. a) BUSCO genome completeness assessment of the 3 strains. The white text shows the completeness score for each assembly. b) Venn diagram of orthologous proteins shared or unique to the 3 strains. c) Whole genome alignment with ProgressiveMauve. Colored blocks indicate regions of high similarity. Red vertical lines designate a new contig in the genome assembly. Blocks below the center line are aligned sequences in the reverse direction. White vertical lines within blocks represent the localized areas that have not aligned. d) Word cloud of the top BLASTp descriptions for the unique KTP proteins. e) Number of BLASTp hits for the unique KTP proteins according to the top strain found. f) Ethanol production of *S. cerevisiae* KTP after 30 h of fermentation in YP with either 10% or 20% glucose.

After classification, we compared the *S. cerevisiae* KTP genome to the probiotic strain *S. boulardii* unique28 and the laboratory strain *S. cerevisiae* S288c. Each genome used in the comparison is nearly complete as estimated by BUSCO. The *S. cerevisiae* KTP assembly has 99.5% of single-copy BUSCOs, while the *S. cerevisiae* S288c assembly has 99.4%, and the *S. boulardii* unique28 assembly has 99.2% (Fig. 3a). We then analyzed orthologs using the protein clustering algorithm OrthoVenn2. We found 5081 orthologs shared among the three yeast strains and KTP had 43 unique sequences, S288c had 42 unique sequences, and unique28 had 8 unique sequences (Fig. 3b). We then compared the genome structures of the three strains using progressive Mauve 2.3.1. These analyses show that the nucleotide alignment of these three strains is very similar (represented as colored blocks in Fig. 3c) and support the argument that *S. boulardii* is a strain of *S. cerevisiae.* Furthermore, the alignment also shows that the genome configuration of *S. cerevisiae* KTP is more like S288c than *S. boulardii* unique28—particularly the pink block beginning at 6 MB is truncated only in *S. b.* unique28 (Fig. 3c).

Unique orthologs are of particular interest because they could be the reason *S. cerevisiae*, KTP exhibits probiotic effects not seen in S288c. Therefore, to classify these orthologs we performed BLASTp of the unique proteins in KTP. This analysis produced many hits across several species of yeasts. The top five gene description hits were heat shock protein Ssb1, elongation factor 1-alpha, amidase, Ato3p, and glyceraldehyde 3-phosphate dehydrogenase (Supplementary Table 2). These were compiled into a word cloud to show the number of hits for each gene (Fig. 3d). For Ssb1, which had five hits, we analyzed whether this was an assembly artifact, but each gene had read coverage consistent with the rest of the genome, ruling out assembly artifacts (Supplementary Fig. 1). Ssb1 is member of *HSP70* family, which are highly abundant heat shock proteins unique to fungi (Dombek *et al*. 2004). The other genes are associated in translation and central carbon metabolism. This indicates that while its genome structure may be similar to S288c, KTP could have key differences in tolerance, gene expression, and metabolism that contribute to the probiotic effect.

We further sorted the BLASTp hits of the unique genes by species, showing that many of the unique proteins are typically shared among wine yeasts such as *S. cerevisiae* AWRI1631, followed by two *Candida* species, and also *S. cerevisiae,* strains CEN.PK113-7D, YJM989 and RM11-1a (Fig. 3e).

An ethanol concentration greater than eight percent (>8%) is a key parameter for a yeast strain to be used in winemaking (Fleet 2008) and *S. cerevisiae* AWRI1631 produces 12% ethanol when grown in medium containing 20% glucose (Contreras *et al*. 2014). Therefore, we measured the ethanol content of *S. cerevisiae* KTP. We found that it produces 5.58 ± 0.09% and 9.07 ± 0.92% ethanol in medium containing 10 and 20% glucose, respectively (Fig. 3f). This result suggests that *S. cerevisiae* KTP is suitable for alcohol fermentation though that is not the focus of our study.

### *S. cerevisiae* KTP and *S. boulardii* share genes implicated in probiotic phenotypes

**Fig. 4.**
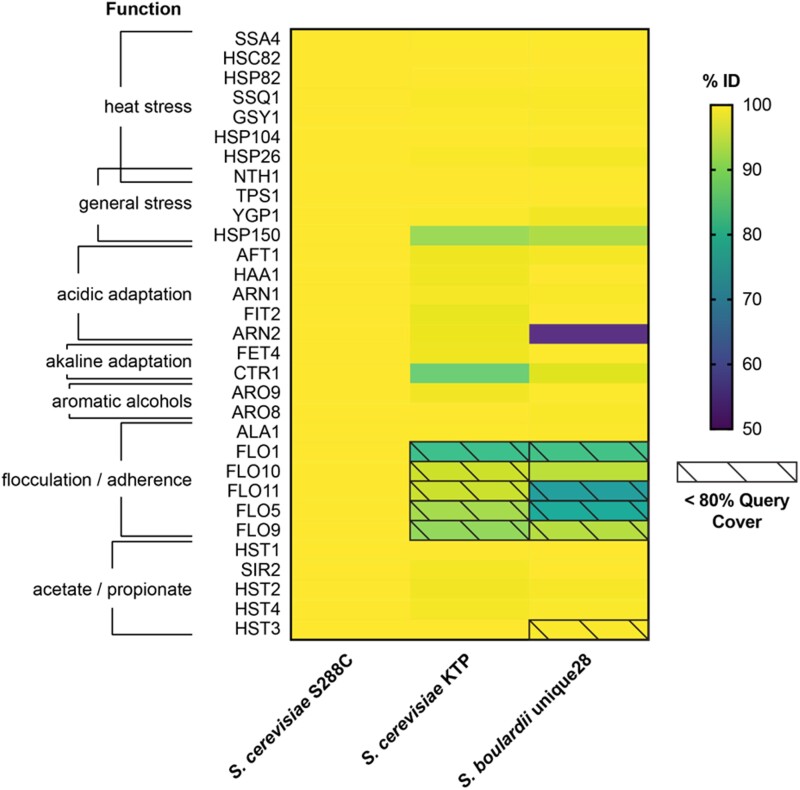
Percent identity to proteins implicated in probiotic phenotypes. BLASTp hits from the *S. cerevisiae* KTP and *S. boulardii* unique28 strains with less than 80% query coverage are shown with black diagonal lines.

Several probiotic phenotypes have known genotypes. These include tolerance, adherence, and metabolite biosynthesis. The conditions of the gastrointestinal tract require tolerance to pH, both acidic and alkaline, as well as tolerance to a temperature of 37°C. Adherence to intestinal epithelial cells is necessary because the natural flow of material through the gastrointestinal tract can quickly clear non-adherent microbes. Biosynthesis of certain metabolites like aromatic alcohols and short chain fatty acids has been associated with several functional attributes in probiotic microbes. Particularly, acetate and propionate can decrease inflammation of the colon, control the secretory activity of gut by modulating enteric nervous system, and improve gut immunity (Koh *et al*. 2016). Further, the aromatic alcohols tryptophol and phenylethanol were shown to inhibit filamentation of *C. albicans* (Kunyeit *et al*. 2021). It has already been shown that *S. cerevisiae* KTP exhibits pH and heat tolerance as well as adherence to intestinal cells (Lohith and Anu-Appaiah 2018). Therefore, we compiled a list of 31 genes that have been implicated in stress tolerance, adherence, and biosynthesis and compared these genes across *S. cerevisiae* KTP, *S. cerevisiae* S288c, and *S. boulardii* unique28 (Fig. 4).

The microenvironment of the human gastrointestinal presents conditions that inhibit growth of microorganisms. However, several microorganisms, including *S. cerevisiae*, can survive adverse conditions of the gut by regulating genes that modulate their response to stresses such as extreme temperature, osmolarity, and availability of key micronutrients such as essential trace elements. We identified 18 genes implicated in heat and pH tolerance. The heat response genes included *HSP26*, *SSA4*, *HSP82*, *HSC82, HSP104*, and *GSY1*, the general stress regulators *TPS1* and *NTH1*, and the long-term heat stress response gene *SSQ1* (Auesukaree *et al*. 2012; Chamnipa *et al*. 2018). The acidic pH tolerance genes included were stress response genes *YGP1* and *HSP150*, the metal metabolism genes *FIT2*, *ARN1*, and *ARN2* (Kawahata *et al*. 2006), and *AFT1* and *HAA1* (Haitani *et al*. 2012). The alkaline pH tolerance genes included were *FET4* and *CTR1* (Serrano *et al*. 2004) (Fig. 4). Large scale studies have identified these genes to be involved in the regulation of general stress responses including metal homeostasis (Yun *et al*. 2000a, 2000b). We believe that these genes likely play a role in modulating the adaptation of food-derived yeasts in the human gastrointestinal tract.

We identified 6 genes involved in adherence. These primarily consisted of the *FLO* family—*FLO1*, *FLO5*, *FLO9*, *FLO10*, and *FLO11—*that encode for cell-wall associated surface proteins and regulate the cell surface properties of *S. cerevisiae* in genetic and epigenetic level (Halme *et al*. 2004; Hope and Dunham 2014). *ALA1* was also included because it has been implicated in the ability to bind extracellular matrices (Gaur and Klotz 1997). The *FLO* family is of particular interest because these genes have been shown to have a large impact on probiotic characteristics. *FLO1*, *FLO5*, *FLO9*, and *FLO10* are responsible for cell-to-cell adhesion as well as adhesion to abiotic surfaces in *S. cerevisiae* (Teunissen and Steensma 1995). *FLO1*, *FLO5* and *FLO9* are important for yeast biofilm formation (Yang *et al*. 2018). *FLO11* is involved in adhesion to agar and abiotic surfaces, sliding motility, filament formation, invasive growth, and substrate adhesion (Guo *et al*. 2000; Halme *et al*. 2004; Bayly *et al*. 2005). Higher expression of *FLO1*, *FLO5* and *FLO11* in *S. cerevisiae* has also been shown to enhance thermotolerance and viability (Vergara-Alvarez *et al*. 2019) (Fig. 4).

We identified 7 genes involved in metabolic pathways relevant to probiotic yeasts. The genes included from acetate and propionate metabolism were *SIR2*, *HST1*, *HST2*, *HST3*, and *HST4* (Starai *et al*. 2003). The genes *ARO8* and *ARO9* were included because they participated in aromatic alcohol biosynthesis (tryptophol and phenylethanol) (Chen and Fink 2006) (Fig. 4).

Each of the 32 genes were then found in *S. cerevisiae* KTP, *S. cerevisiae* S288c, and *S. boulardii* unique 28 using BLASTp. The percent identity of the top protein hit to the query is shown in Fig. 4. Interestingly, both KTP and *S. boulardii* unique28 have less than 80% query coverage for *FLO1, FLO10, FLO5, FLO9, and FLO11*. The observed differences are not due to assembly, as read coverage is consistent across these five genes (Supplementary Fig. 2). Protein sequence alignment of the KTP and S288c sequences for each are shown in Supplementary Figs. 3, 4, 5, 6 and 7. In addition to the *FLO* family, the genes encoding the heat shock protein *HSP150* and the alkaline adaptation gene *CTR1* had a lower query coverage; however, these may be due to assembly artifacts (Supplementary Fig. 2).

### Conclusion

These results show that WGS is vital for accurate taxonomic classification and genome analysis of probiotic yeasts. Using an accurate genome assembly, we were able to determine that *S. cerevisiae* KTP is from a different *Saccharomyces* lineage than *S. boulardii*, yet they both share mutations in flocculation genes, suggesting at a convergent evolutionary strategy for probiotic mechanisms. We also showed that *S. cerevisiae* KTP shares several gene classes with wine yeasts and produces ~8% ethanol. We also showed that *I. occidentalis* ApC is from a branch of nonconventional yeasts with few probiotic strains recognized. Its genome is significantly different from the other probiotic strains, suggesting it may have a different probiotic strategy. This is an area for future probiotic research as more genomes of non-*Saccharomyces* probiotic yeasts become available. Overall, this study provides a blueprint for WGS of promising yeast strains that could have medicinal or industrial benefit and demonstrates how analysis of accurate genomic information can yield insight into phenotype-genotype relationships.

## Supplementary Material

jkad093_Supplementary_Data

## Data Availability

*S. cerevisiae* KTP has been assigned the NCBI BioSample accession SAMN30285631, and all data are available under BioProject PRJNA869102. The whole genome assembly can be accessed with accession JANQBH000000000. The raw reads are available at the NCBI Sequence Read Archive (SRA)—nanopore reads are available under accession SRR21031641 and illumina reads are available under accession SRR21031642. *I. occidentalis* has been assigned the NCBI BioSample accession SAMN30286403, and all data are available under BioProject PRJNA869107. The whole genome assembly can be accessed with accession JANQBI000000000. The raw reads are available at the NCBI SRA—nanopore reads are available under accession SRR21034916 and illumina reads are available under accession SRR21034917. Supplemental material available at G3 online.
