# Supplementary material for: Genetic basis for probiotic yeast phenotypes revealed by nanopore sequencing
Source: G3 (Bethesda). 2023 Apr 27;13(8):jkad093. doi: 10.1093/g3journal/jkad093 (PMC10411601; doi:10.1093/g3journal/jkad093)
Supplement: jkad093_Supplementary_Data [file jkad093_supplementary_data.docx]

**Supplementary figure and table**

**Table S1**: Genome assembly statistics, number of protein-coding genes, and top 10 ITS BLAST species results for the KTP and ApC strains.

| **Strain** | **Origin** | **No. of contigs** | **N50 (Mbp)** | **Assembly length (Mbp)** | **GC (%)** | **No. of protein coding genes** | **Top 10 ITS BLAST species** |
| --- | --- | --- | --- | --- | --- | --- | --- |
| KTP | Coconut toddy | 28 | 0.82 | 12.23 | 38.16 | 6159 | *Saccharomyces cerevisiae* |
| ApC | Fermented apple juice | 7 | 2.85 | 11.85 | 40.17 | 4876 | uncultured Saccharomycetes, *Pichia kudriavzevii,* Saccharomycetes sp.,  *Pichia cecembensis*, *Pichia occidentalis* |

**Table S2.** Top BLAST hits of genes in *S. cerevisiae* KTP and the top strain from the BLAST results. These results were used to create the word cloud in Figure 3d and the BLAST hit chart in Figure 3e.

| **Gene** | **Top BLAST description** | **Top BLAST strain** |  |
| --- | --- | --- | --- |
| KTP\|scaffold 20.g4960.t1 | amidase | *Saccharomyces cerevisiae* AWRI796 | |
| KTP\|scaffold 5.g4954.t1 | amidase | *Saccharomyces cerevisiae* RM11-1a | |
| KTP\|scaffold 15.g1054.t1 | Ato3p | *Saccharomyces cerevisiae* Kyokai no. 7 | |
| KTP\|scaffold 5.g4953.t1 | Ato3p | *Saccharomyces cerevisiae* RM11-1a | |
| KTP\|scaffold 4.g5622.t1 | elongation factor 1-alpha | *Saccharomyces cerevisiae* AWRI1631 | |
| KTP\|scaffold 6.g5922.t1 | elongation factor 1-alpha | *Saccharomyces cerevisiae* AWRI1631 | |
| KTP\|scaffold 7.g220.t1 | glyceraldehyde-3-phosphate dehydrogenase | *Clavispora lusitaniae* ATCC 42720 | |
| KTP\|scaffold 3.g1504.t1 | glyceraldehyde-3-phosphate dehydrogenase | *Clavispora lusitaniae* ATCC 42720 | |
| KTP\|scaffold 16.g618.t1 | heat shock protein SSB1 | *Candida tropicalis* MYA-3404 | |
| KTP\|scaffold 18.g409.t1 | heat shock protein SSB1 | *Clavispora lusitaniae* ATCC 42720 | |
| KTP\|scaffold 16.g617.t1 | heat shock protein SSB1 | *Clavispora lusitaniae* ATCC 42720 | |
| KTP\|scaffold 14.g2433.t1 | heat shock protein SSB1 | *Clavispora lusitaniae* ATCC 42720 | |
| KTP\|scaffold 1.g2375.t1 | heat shock protein SSB1 | *Saccharomyces* *cerevisiae* AWRI1631 | |
| KTP\|scaffold 25.g3.t1 |  | *Candida albicans* SC5314 | |
| KTP\|scaffold 1.g1765.t1 |  | *Candida albicans* SC5314 | |
| KTP\|scaffold 1.g2097.t1 |  | *Candida albicans* SC5314 | |
| KTP\|scaffold 1.g2100.t1 |  | *Candida albicans* SC5314 | |
| KTP\|scaffold 1.g2104.t1 |  | *Candida albicans* SC5314 | |
| KTP\|scaffold 1.g2107.t1 |  | *Candida albicans* SC5314 | |
| KTP\|scaffold 1.g2110.t1 |  | *Candida albicans* SC5314 | |
| KTP\|scaffold 1.g2123.t1 |  | *Candida albicans* SC5314 | |
| KTP\|scaffold 2.g3508.t1 |  | *Candida albicans* SC5314 | |
| KTP\|scaffold 6.g5991.t1 |  | *Candida albicans* SC5314 | |
| KTP\|scaffold 3.g1174.t1 |  | *Clavispora lusitaniae*  ATCC 42720 | |
| KTP\|scaffold 1.g1846.t1 |  | *Clavispora lusitaniae*  ATCC 42720 | |
| KTP\|scaffold 1.g1988.t1 |  | *Clavispora lusitaniae*  ATCC 42720 | |
| KTP\|scaffold 12.g4370.t1 |  | *Clavispora lusitaniae*  ATCC 42720 | |
| KTP\|scaffold 2.g3248.t1 |  | *Kazachstania naganishii* CBS 8797 | |
| KTP\|scaffold 4.g5262.t1 |  | *Kazachstania naganishii* CBS 8797 | |
| KTP\|scaffold 10.g4212.t1 |  | *Pneumocystis jirovecii* | |
| KTP\|scaffold 5.g4545.t1 |  | *Pneumocystis jirovecii* | |
| KTP\|scaffold 18.g481.t1 |  | *Saccharomyces cerevisiae*AWRI1631 | |
| KTP\|scaffold 13.g714.t1 |  | *Saccharomyces cerevisiae*AWRI1631 | |
| KTP\|scaffold 3.g1568.t1 |  | *Saccharomyces cerevisiae*AWRI1631 | |
| KTP\|scaffold 1.g2082.t1 |  | *Saccharomyces cerevisiae*AWRI1631 | |
| KTP\|scaffold 14.g2503.t1 |  | *Saccharomyces cerevisiae*AWRI1631 | |
| KTP\|scaffold 14.g2599.t1 |  | *Saccharomyces cerevisiae*AWRI1631 | |
| KTP\|scaffold 8.g2660.t1 |  | *Saccharomyces cerevisiae*AWRI1631 | |
| KTP\|scaffold 2.g3415.t1 |  | *Saccharomyces cerevisiae*AWRI1631 | |
| KTP\|scaffold 10.g4175.t1 |  | *Saccharomyces cerevisiae*AWRI1631 | |
| KTP\|scaffold 4.g5407.t1 |  | *Saccharomyces cerevisiae*AWRI1631 | |
| KTP\|scaffold 6.g5962.t1 |  | *Saccharomyces cerevisiae*AWRI1631 | |
| KTP\|scaffold 2.g3247.t1 |  | *Saccharomyces cerevisiae*CEN.PK113-7D | |
| KTP\|scaffold 2.g3249.t1 |  | *Saccharomyces cerevisiae*CEN.PK113-7D | |
| KTP\|scaffold 4.g5261.t1 |  | *Saccharomyces cerevisiae*CEN.PK113-7D | |
| KTP\|scaffold 4.g5263.t1 |  | Saccharomyces cerevisiae CEN.PK113-7D | |
| KTP\|scaffold 15.g1055.t1 |  | *Saccharomyces cerevisiae*RM11-1a | |
| KTP\|scaffold 18.g478.t1 |  | *Saccharomyces cerevisiae*YJM789 | |
| KTP\|scaffold 13.g716.t1 |  | *Saccharomyces cerevisiae*YJM789 | |
| KTP\|scaffold 6.g6151.t1 |  | *Saccharomyces cerevisiae*YJM789 | |
| KTP\|scaffold 3.g1359.t1 |  | *Spathaspora passalidarum* NRRL Y-27907 | |
| KTP\|scaffold 8.g2891.t1 |  | *Spathaspora passalidarum* NRRL Y-27907 | |


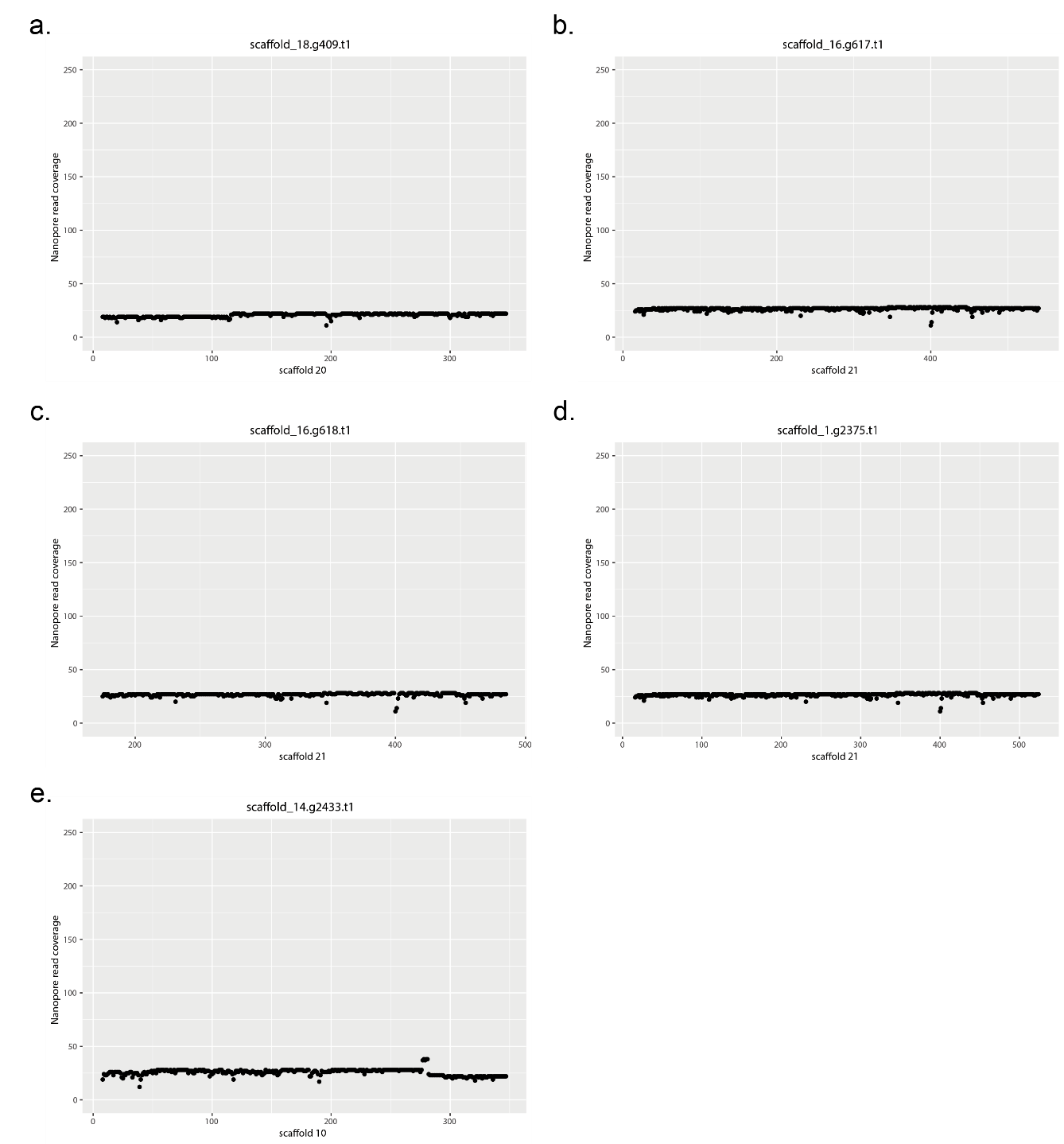


**Figure S1.** Nanopore read coverage of the top BLAST hits in Table S2. Read coverage is consistent with the average read coverage across the entire genome, indicating these truly are duplicated in *S. cerevisiae* KTP. **a.** Read coverage of SSB1 hit g409 on scaffold 20. **b.** Read coverage of SSB1 hit g617 on scaffold 21. **c.** Read coverage of SSB1 hit g618 on scaffold 21. **d.** Read coverage of SSB1 hit g2375 on scaffold 21. **e.** Read coverage of SSB1 hit g2433 on scaffold 10.


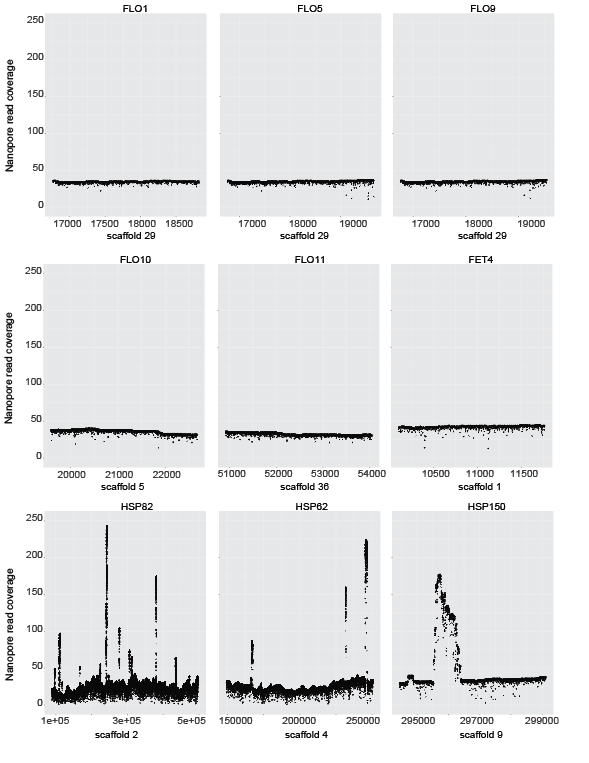


**Figure S2.** Nanopore read coverage of the nine probiotic genes found to be divergent in *S. cerevisiae* KTP. Read coverage for the FLO genes are consistent with the average read coverage across the entire genome, indicating that the differences found are not due to assembly difficulty of the FLO genes. Rather, the HSP genes have regions of high read coverage, indicating that the differences observed may be due to assembly.

**
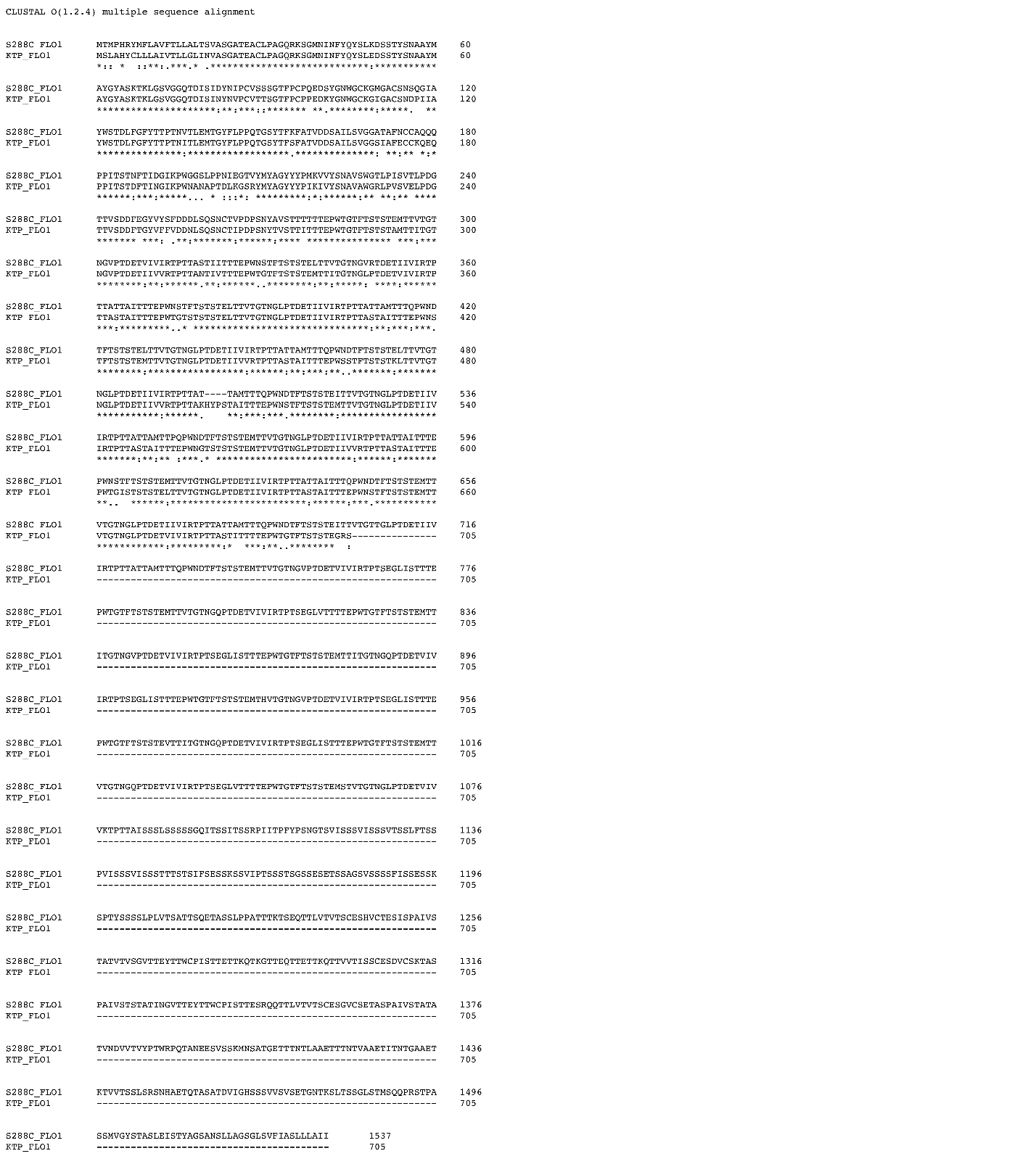
**

**Figure S3.** FLO1 protein sequence alignment between *S. cerevisiae* S288C and KTP.

**
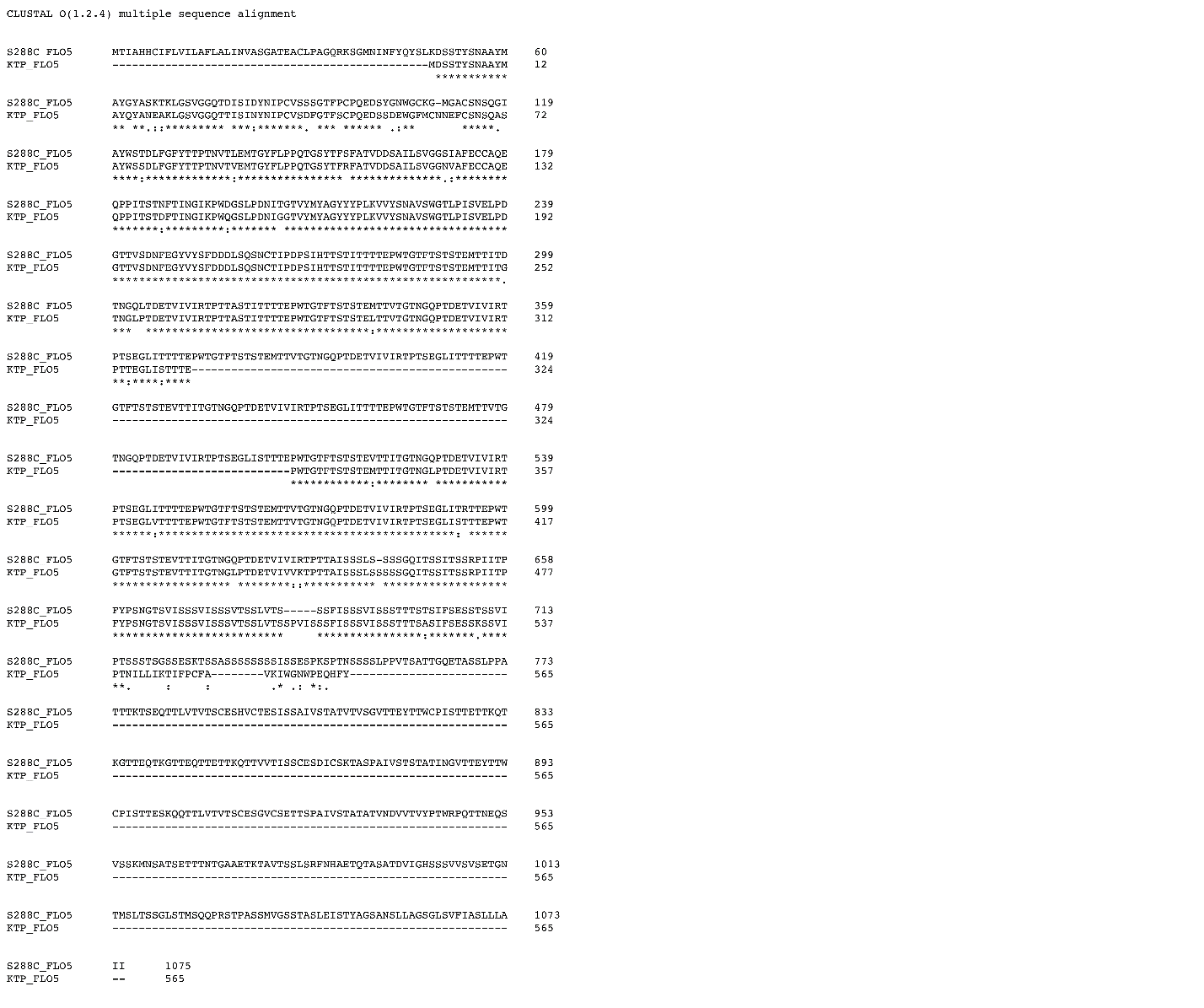
**

**Figure S4.** FLO5 protein sequence alignment between *S. cerevisiae* S288C and KTP.

**
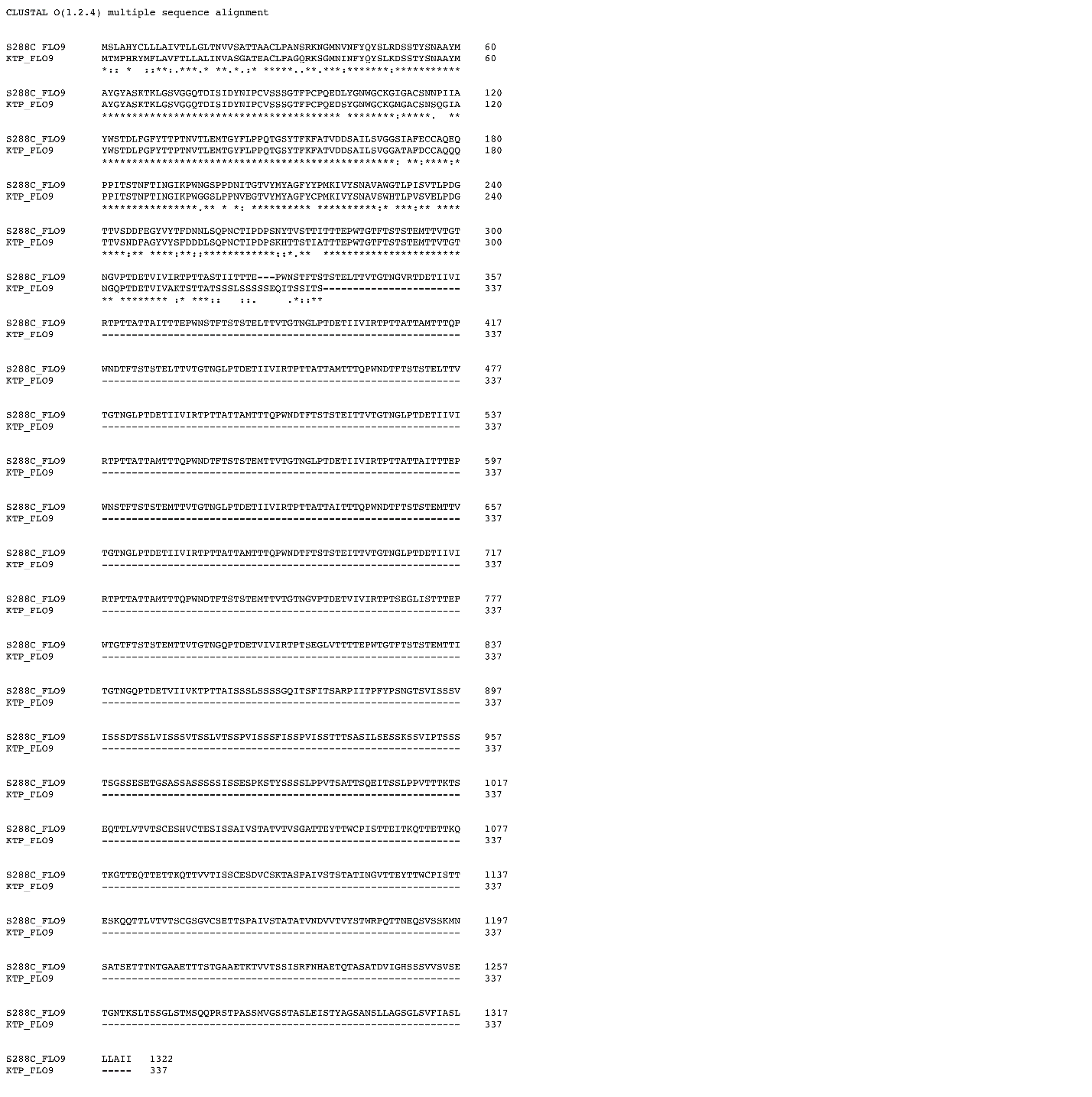
**

**Figure S5.** FLO9 protein sequence alignment between *S. cerevisiae* S288C and KTP.

**
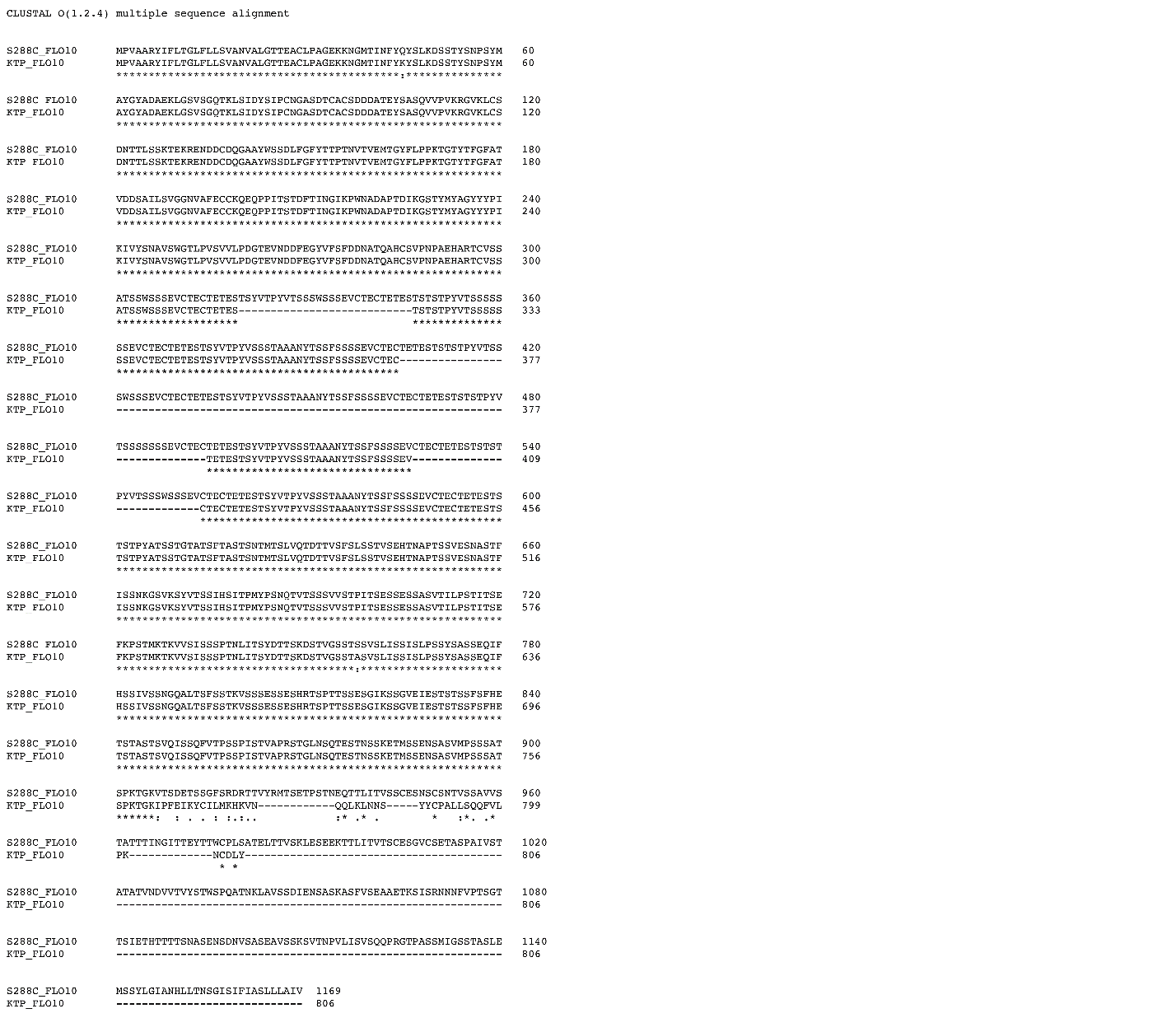
**

**Figure S6.** FLO10 protein sequence alignment between *S. cerevisiae* S288C and KTP.

**
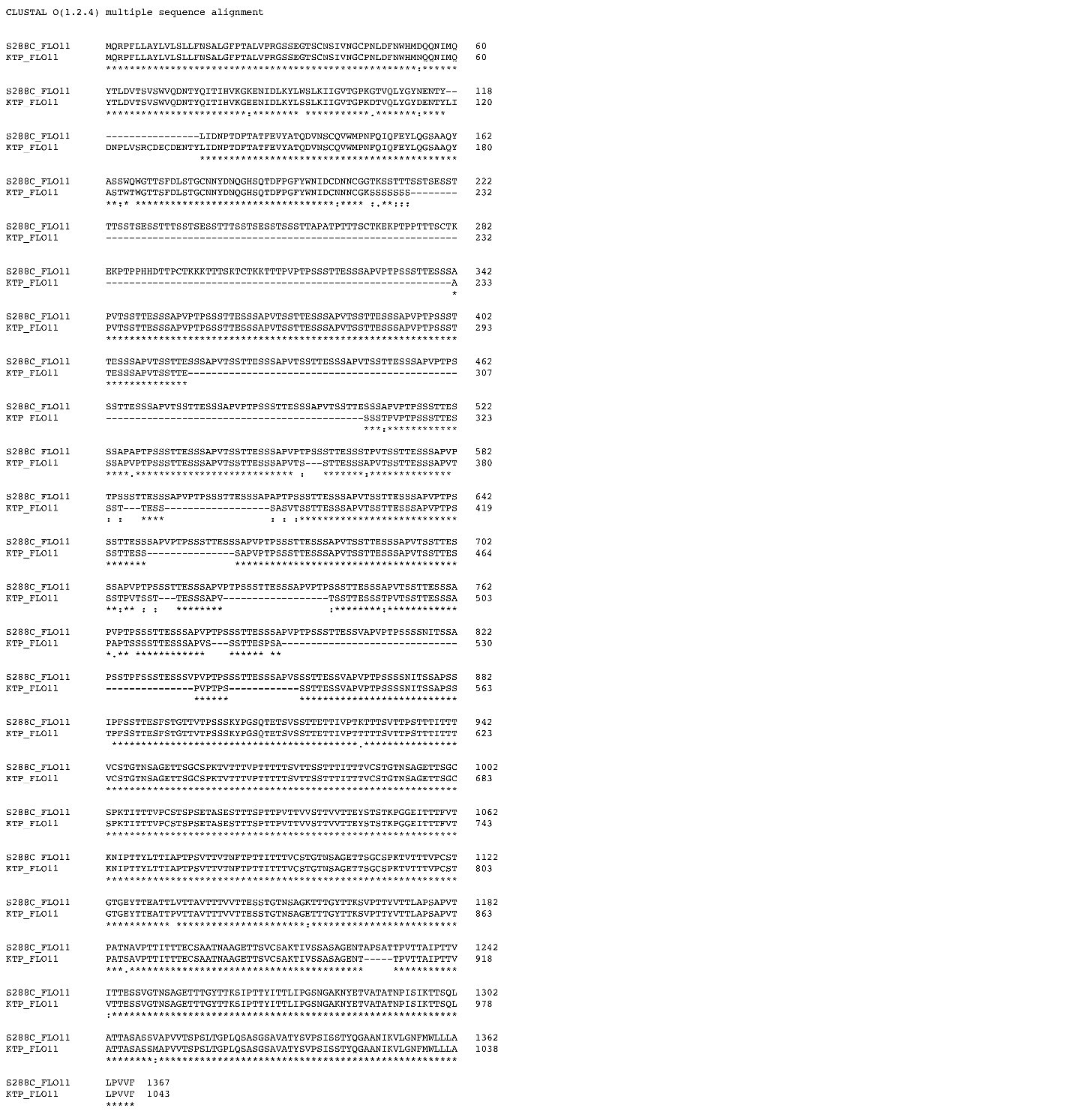
**

**Figure S7.** FLO11 protein sequence alignment between *S. cerevisiae* S288C and KTP.
